# (Mis)Information on Digital Platforms: Quantitative and Qualitative Analysis of Content From Twitter and Sina Weibo in the COVID-19 Pandemic

**DOI:** 10.2196/31793

**Published:** 2022-02-24

**Authors:** Sarah Kreps, Julie George, Noah Watson, Gloria Cai, Keyi Ding

**Affiliations:** 1 Department of Government Cornell University Ithaca, NY United States; 2 Department of Information Science Cornell University Ithaca, NY United States; 3 Department of Computer Science Cornell University Ithaca, NY United States; 4 Department of Music Cornell University Ithaca, NY United States; 5 Department of Asian Studies Cornell University Ithaca, NY United States

**Keywords:** internet, social media, misinformation, COVID-19, Twitter, Weibo, prevalence, discourse, content, communication, public health, context, content analysis

## Abstract

**Background:**

Misinformation about COVID-19 on social media has presented challenges to public health authorities during the pandemic. This paper leverages qualitative and quantitative content analysis on cross-platform, cross-national discourse and misinformation in the context of COVID-19. Specifically, we investigated COVID-19-related content on Twitter and Sina Weibo—the largest microblogging sites in the United States and China, respectively.

**Objective:**

Using data from 2 prominent microblogging platform, Twitter, based in the United States, and Sina Weibo, based in China, we compared the content and relative prevalence of misinformation to better understand public discourse of public health issues across social media and cultural contexts.

**Methods:**

A total of 3,579,575 posts were scraped from both Sina Weibo and Twitter, focusing on content from January 30, 2020, within 24 hours of when WHO declared COVID-19 a “public health emergency of international concern,” and a week later, on February 6, 2020. We examined how the use and engagement measured by keyword frequencies and hashtags differ across the 2 platforms. A 1% random sample of tweets that contained both the English keywords “coronavirus” and “covid-19” and the equivalent Chinese characters was extracted and analyzed based on changes in the frequencies of keywords and hashtags and the Viterbi algorithm. We manually coded a random selection of 5%-7% of the content to identify misinformation on each platform and compared posts using the WHO fact-check page to adjudicate accuracy of content.

**Results:**

Both platforms posted about the outbreak and transmission, but posts on Sina Weibo were less likely to reference topics such as WHO, Hong Kong, and death and more likely to cite themes of resisting, fighting, and cheering against coronavirus. Misinformation constituted 1.1% of Twitter content and 0.3% of Sina Weibo content—almost 4 times as much on Twitter compared to Sina Weibo.

**Conclusions:**

Quantitative and qualitative analysis of content on both platforms points to lower degrees of misinformation, more content designed to bolster morale, and less reference to topics such as WHO, death, and Hong Kong on Sina Weibo than on Twitter.

## Introduction

As the COVID-19 pandemic began to emerge in the early weeks of January 2020, information about the mechanism, location, and speed of transmission, as well as the array of government actions to stop the spread of the virus, was limited. Individuals worldwide turned to social media for information, spending an average of 82 minutes per day on social media compared to 75 minutes a year earlier. Twitter, as 1 observer put it, “especially shone as a real-time news source” of breaking news and analysis about the virus [1]. In the United States, in the first quarter of 2020, Twitter’s daily user figures were 24% higher than for the same period a year earlier [2]. In China, individuals turned to their equivalent of Twitter, Sina Weibo (referred to here as Weibo), to learn about the virus and exchange concerns.

Although the role of social media, such as Twitter, has received considerable scrutiny in political contexts, such as conflict, revolts, and elections [3], it had, until recently, been less scrutinized in a public health context [4]. Twitter emerged as a platform for discussion about the Ebola virus in 2014 [5], with studies showing that many tweets were inaccurate and wildly speculative compared to those that were scientifically accurate. Individuals also took to Twitter activity in 2015 and 2016 to discuss virus transmission, treatment, and symptoms, providing a measure of public health surveillance to track and predict the Zika virus but also amplifying rumors and misinformation, defined as incorrect information that is not intentionally false [6] about the virus [7]. The proliferation of misinformation, even when harmless, can result in serious social and lethal health consequences in the context of pandemics [8]. As the number of Twitter users has grown in the intervening years since Ebola and Zika, so has the centrality of Twitter in the context of the recent pandemic to the extent that COVID-19 has been referred to as the “Twitter Pandemic” because of its role in distributing medical information and misinformation [9]. For example, a March 12, 2020, tweet falsely claimed that Costco had recalled toilet paper it thought was contaminated with COVID-19, including old video repurposed to support the false claim [10].

Weibo has occupied an analogous space as Twitter in the Chinese context [11]. The Chinese microblogging platform was launched by Sina Corporation in August 2009, after Twitter was blocked in China earlier that year due to anniversary protests at Tiananmen Square [12]. As King et al [13] note, individuals in China have access to a number of different social media platforms, but Weibo is a widely used microblogging platform, with over 430 million monthly active users, a large proportion of China’s population [14], compared to Twitter’s 326 million users a month. The platform has been criticized in Western media outlets for limiting free speech [15]. King et al [16] find a tendency on Chinese social media platforms not necessarily to censor criticism of the government altogether but more to avoid controversial issues that might have an unsettling impact on social order and to steer toward more benign topics less likely to stir the public. Alternatively, and in the particular context of COVID-19 content, Lu et al [17] suggest that China permitted criticism of the regime but that those criticisms were matched by statements of support for the progress and positive outcomes associated with the epidemic. Indeed, criticisms “targeted at the government for perceived lack of action, incompetence, and wrongdoing” [17] were complemented almost exactly proportionately with bursts of support for the regime. Thus, previous research suggests the possibility that there will be a relative dearth of subjects on Weibo that might rouse the public, favoring instead either anodyne content or a complementarity intending to balance criticisms with support.

Further, research has provided evidence in other public health contexts about the comparatively higher amounts of misinformation on Twitter compared to Weibo. In a study of misinformation surrounding the Ebola epidemic in 2014-2015 comparing Weibo and Twitter, Fung et al [18] found that the amount of misinformation is low for each platform and does not exhibit meaningful differences across platforms. Relatedly, the authors found that most content focuses on outbreak-related news, Ebola health communication, and responses on both social media platforms. Weibo did, however, emphasize favorable Chinese government behavior—sending relief to Guinea—compared to Twitter. Although a useful comparative study for Ebola, the previous study is unlikely an appropriate analogy for the COVID-19 epidemic because of the coronavirus’s origins in China, which implicated the Chinese government, thereby creating the type of setting where China might have more incentives to shape a particular narrative away from controversial issues [18].

More recently, Rodriguez et al [19] compared COVID-19 misinformation on Weibo and Twitter, although subsetted to a small fraction of total posts by using a keyword search of “coronavirus,” which yielded fewer than 2000 social media posts during their period of study. In addition, the authors extracted an equal number of tweets and Weibo posts, which does not account for the differing sample sizes of users across the 2 platforms. Further, they limited the analysis to just 2 days in February 2020, specifically February 6 and 7, when Dr Li Wenliang, who raised the alarm about coronavirus in China, passed away. The authors did, however, find more misinformation on Twitter than on Weibo. In the following section, we describe our method for studying COVID-19 content across the 2 platforms by way of understanding both the type of discourse and also the potential exposure to misinformation on both Weibo and Twitter. The objective of this study is to compare COVID-19-related information and the relative prevalence of misinformation to further understand public discourse across social media and cultural contexts.

## Methods

### Study Design

To compare content related to COVID-19 on Twitter and Weibo, including misinformation, we studied 3,579,575 posts from both Weibo and Twitter—2,344,332 (65.49%) tweets on Twitter and 1,235,243 (34.51%) posts on Weibo—focusing on content from January 30, 2020, when the World Health Organization (WHO) declared COVID-19 a “public health emergency of international concern,” and February 6, 2020. We then compared top keywords, hashtags, and misinformation (guided by WHO’s COVID-19 misinformation website [8]) for both platforms.

### Ethics Statement

We registered an academic research application for Twitter’s Application Programming Interface (API) in December 2020, which allowed us to search for specific keywords and key dates and obtain Twitter users’ publicly available tweets across 2 different batches of posts. Because the posts were made publicly, they were exempt from requiring institutional review board approval. Moreover, our study only included secondary data analysis of publicly available information and deidentified personal individuals’ information. The Twitter API allows academic researchers with specific research objectives to obtain precise, complete, and unbiased data, while protecting the security and privacy of people on Twitter and the developer platform.

### Data Collection and Analysis

With respect to Weibo, we used a large-scale COVID-19 social media data set that includes a total of over 40 million Weibo posts [20]. The data set, Weibo-COV, covers posts from December 1, 2019, to April 30, 2020, and contains variables such as location, repost network, post time, and interaction information. To obtain access to the Weibo-COV corpus, we submitted a research application that outlined the objectives of our study to the authors of the data set and received approval. All posts were in Mandarin and therefore accessible to the Mandarin speakers on the research team. Three members of the research team are fluent in reading and speaking Mandarin, 2 of whom are native Mandarin speakers.

Drawing on the approach of Fung et al [21], we compared a 1% random sample of Twitter and Weibo content in the early stages of the pandemic. A random selection of 1%, given the millions of total posts we used, yielded more than 35,000 total posts and is both likely to be representative of content but also manageable from an analysis perspective. The first batch of posts consisted of a 1% random sample of Tweets and Weibo posts created within 24 hours of the WHO declaration that the 2019-SARS-CoV-2 outbreak was a “public health emergency of international concern” (January 30, 2020). The second batch was a 1% random sample of Tweets and Weibo posts created 1 week after the WHO declaration (February 6, 2020), both searching English keywords “coronavirus” and “covid-19” and the Chinese words “新冠,” “新型冠状病毒,” and “疫情.” These 2 windows provide insights into how social media users react, discuss, and interact with content, potentially content that includes purposefully misleading or inadvertently factually incorrect information. Furthermore, the 1-week period allowed us to compare whether there is substantial content moderation on alternative health information across the 2 platforms.

Due to the complexity of the Chinese text, we then segmented the text into phrases using the Viterbi algorithm [18]. The Viterbi algorithm is a dynamic programming algorithm for identifying the most likely sequence of hidden states, otherwise known as the Viterbi path, that results in a sequence of observed events. In this case, the algorithm helped us with segmenting Chinese words and phrases for readability. We recorded the contents and time of posting for each microblog post in a comma-separated file.

We also conducted relative risk (RR) analysis, which emphasized the direction of the relative frequency of keywords and hashtags across the 2 batches. Items that had an RR of greater than 1 were considered trending, whereas a fading item was identified by a number less than 1. To calculate the RR for a keyword or hashtag, we used the following equation:

RR_i_ = P_iBatch 2_/P_iBatch 1_

The numerator denotes the probability of tweets/Weibo posts with item i in batch 2, whereas the denominator denotes the probability of tweets/Weibo posts with item i in batch 1.

After computing the RR, we manually coded a random selection of 5%-7% of the social media posts, which comprised the initial 1% random samples, following Fung et al [18]. We then assigned a random number between 0 and 100 for the tweets and posts; if a post was assigned a number of 5 or less, we selected it for manual coding. The proportion of the random numbers was different for each data set, so the manually coded data sets consisted of the following:

- Twitter: batch 1, n=954 (6.1%) of 15,737 tweets; batch 2, n= 448 (5.8%) of 7726 tweets
- Weibo: batch 1, n=279 (5.7%) of 4914 posts; batch 2, n=441 (5.9%) of 7439 posts

Within each selected sample, we categorized the posts into English/Chinese posts and non-English/non-Chinese posts and excluded the latter. Using Fung et al’s [21] classification of topics, 3 coders read and classified the content. Each coder first independently reviewed the tweets and Weibo posts and identified them by various categories. After completing this step, the research team then recoded the content to examine and verify whether category decisions aligned across the tweets and posts. Finally, all manual coding efforts were checked by the lead coder for a wide-ranging review and deconfliction. We also included a few unique categories that relate to COVID-19 in our classification, similar to Fung et al’s [18] categorization for Ebola-related content (eg, “News of a Case of Someone Spreading Rumor of ‘Ebola in Pudong’ Being Detained by Police,” “Assistance to Guinea-Chinese Medical Team Departure for Guinea”). This decision was made because a portion of the tweets and Weibo posts did not fit into the original categories that Fung et al [18] had designed, but we deemed important, substantial in number, and particular to the COVID-19 situation (eg, “Cheer on,” “Dali,” “News About Li Wenliang”). In this manner, we provide a comprehensive, multifaceted review of Weibo and Twitter content during these 2 pivotal dates.

In addition, we determined whether tweets and Weibo posts contained sources of misinformation through our manually coding and categorizing of the randomly selected subdata sets. Specifically, we manually categorized microblog contents under different themes to identify the information and misinformation, using the WHO fact-check website to adjudicate accuracy calls [8]. Importantly, WHO has communicated with more than 50 digital companies and social media platforms to safeguard that science-based health messages from the organization or other official sources appear first when individuals search for information concerning COVID-19. Its Mythbusters page includes the refutation of falsehoods, such as assertions that water or alcohol can protect against COVID-19 or that the virus cannot spread in humid climates.

## Results

### Data Analysis

Our analysis suggested that Twitter has far more posts centered on the virus, a total of 2,344,322 tweets across the 2 batches, despite the virus being more concentrated in China than in the United States at the time, compared to 1,235,243 Weibo posts across the 2 batches, as illustrated in the number of coronavirus-related posts retrieved shown in Tables 1 and 2. As Table 1 shows in more detail, a number of keywords appear across both Weibo and Twitter batches: “coronavirus,” “Wuhan,” and even “Li Wenliang.” Dr Li Wenliang was a Chinese ophthalmologist known for raising awareness of the early COVID-19 outbreak in Wuhan. Dr Li Wenliang passed away on February 7, 2020, 1 week after the WHO announcement and date of our second batch.

**Table 1:** Top 20 most frequent words on Weibo and Twitter.

Results^a^	Weibo (N=1,235,243)		Twitter (N=2,344,322)	
	Batch 1	Batch 2	Batch 1	Batch 2
COVID-19 posts/tweets retrieved (raw data), n (%)	491,353 (39.78)	743,890 (60.22)	1,572,928 (67.09)	771,404 (32.91)
Relevant posts/tweets analyzed (1% random sample), n/N	4914/491,353	7439/743,890	15,737/1,572,928	7726/771,404
Top 20 most frequent keywords	疫情 (epidemic situation)	疫情 (epidemic situation)	Coronavirus	Coronavirus
	理由 (justification)	理由 (justification)	China	China
	肺炎 (pneumonia)	肺炎 (pneumonia)	Health	Wuhan
	冠状病毒 (coronavirus)	口罩 (mask)	Virus	Virus
	武汉 (Wuhan)	大理 (Dali)	Outbreak	Li Wenliang
	新型 (new type)	加油 (to cheer on)	WHO^b^	Doctor
	口罩 (mask)	武汉 (Wuhan)	People	Outbreak
	感染 (infect)	确诊 (diagnose)	Wuhan	People
	加油 (to cheer on)	物资 (goods and materials)	Emergency	Death
	宠物 (pet)	求助 (seek help)	Cases	Cases
	确诊 (diagnose)	冠状病毒 (coronavirus)	Global	Hospital
	医院 (hospital)	新型 (new type)	Public	News
	病例 (case of illness)	征用 (expropriate)	World	World
	医生 (doctor)	防控 (prevent and control)	Confirmed	Public
	抗击 (resist/fight back)	患者 (sufferer)	Spread	Health
	黄冈市 (Huanggang, prefecture-level city in Hubei)	李文亮 (Li Wenliang)	Breaking	Disease
	病毒 (virus)	感染 (infect)	First	Police
	防控 (prevent and control)	信息 (information)	Illness	Epidemic
	隔离 (quarantine)	医院 (hospital)	Travel	Media
	卫健委 (National Health Commission)	抗击 (resist/fight back)	Declared	Infected

^a^Reflects data from 2 cross-sectional samples of Twitter tweets and Chinese microblogs (Weibo) on COVID-19, January 30-31, 2020 (batch 1), and February 6-7, 2020 (batch 2). Keywords and hashtags are used in Twitter and Chinese microblogs for a number of reasons, such as emphasizing the theme of the post.

^b^WHO: World Health Organization.

**Table 2:** Top 10 most frequent hashtags on Weibo and Twitter.

Results^a^	Weibo (N=1,235,243)		Twitter (N=2,344,322)	
	Batch 1	Batch 2	Batch 1	Batch 2
COVID-19 posts/tweets retrieved (raw data), n (%)	491,353 (39.78)	743,890 (60.22)	1,572,928 (67.09)	771,404 (32.91)
Posts/tweets with hashtags (percentage of analyzed posts/tweets, n/N (%)	3418/4914 (69.55)	4528/7439 (60.87)	4982/15,737 (31.66)	2468/7726 (31.94)
Top 10 most frequent hashtags	共同战役 (Fight the pandemic together.)	新型肺炎求助通道开启 (new COVID help channel opened)	Coronavirus	Coronavirus
	武汉加油 (Go Wuhan.)	武汉加油 (Go Wuhan.)	China	China
	最近疫情地图 (latest epidemic map)	抗疫行动 (fighting COVID movement)	2019ncov	Wuhan
	世卫组织称无证据显示宠物会感染 (WHO says there is no evidence that pets can get infected.)	手写加油接力 (Show your support challenge.)	Coronavirusoutbreak	2019ncov
	抗击新型肺炎我们在行动 (We are acting in the fight against COVID.)	李文亮医生去世 (Dr Li Wenliang passed away.)	Wuhan	Coronavirusoutbreak
	黄冈疾控负责人一问三不知 (The leader of Huanggang Disease Control doesn't know anything.)	最新疫情地图 (latest epidemic map)	nCov	wuhancoronavirus
	疫情仍处于扩散阶段 (The epidemic is still in the spreading stage.)	大理欠理了 (Dali messed up.)	Breaking	Liwenliang
	疫情结束后最想吃的东西 (what you want to eat most after the epidemic is over)	肺炎患者求助 (COVID patients ask for help.)	WuhanCoronavirus	HongKong
	新型冠状病毒 (novel coronavirus)	新华锐评 (XinHua/CCP news channel commentary)	PrayforChina	CoronaOutbreak
	河南多地急需医护物资 (Many places in Henan urgently need medical supplies.)	抗击新型肺炎第一线 (the first line of fighting COVID)	HongKong	CoronavirusChina

^a^Top hashtags identified in 2 cross-sectional samples of Twitter tweets and Chinese microblogs (Weibo) on COVID-19, January 30-31, 2020 (batch 1), and February 6-7, 2020 (batch 2).

Despite high areas of overlap between the 2 platforms’ content, Weibo’s content entirely omitted several references that were present on Twitter, including WHO and death. The only reference of WHO on Weibo related to a popular hashtag that underscores the message that pets cannot get infected with COVID-19. In that period, people were not dying in the Twitter-using world, suggesting that individuals writing about death in the Twitter context were referencing the situation in China, and yet “death” was absent from the top words used on Weibo. Weibo instead appeared to reference “pneumonia,” a less acute and potentially survivable medical condition. In particular, we saw that Dali, a city in Southwest China’s Yunnan Province, was also a popular keyword and hashtag in the Weibo analysis due to the public’s reaction over a large controversy [22]. During the 1-week period, Dali intercepted a shipment of masks that was meant for the Chongqing municipality and Huangshi in Central China’s Hubei Province, which was the epicenter of the outbreak. As a result, many Weibo users were angry at the city of Dali for intercepting a shipment of surgical masks that had only 8 confirmed cases of COVID-19, whereas the hard-hit Chongqing municipality had 400 cases. Moreover, the government of Dali had already distributed the boxes of surgical masks and could not retrieve them after Chongqing demanded for the shipment [23]. As for the Twitter analysis, we learned that users are interested in the “global” effects of COVID-19 through posts on travel restrictions, Hong Kong, the overall spread, and WHO.

Beyond excluding some topics, such as WHO, that were common on Twitter, while including topics, such as Dali, that were critical of government officials, the Weibo content also included whole categories of posts that pushed positive themes and were intended to be reassuring compared to the absence of those types of themes being prevalent on Twitter. Keywords from the Weibo content focused on more positive and encouraging messages or themes (eg, “to cheer on” or the hashtag “pray for China”) or empathy (“sufferer”) compared to Twitter keywords (eg, “death”), which did not appear in the Weibo list. Generally, we found that the Weibo analysis included a substantial amount of unified support in “fighting” the COVID-19 pandemic and for health care workers. For example, 1 Weibo post read, “#2020好起来# #抗疫行动# 勤洗手 戴口罩 2020一定会好起来的 加油！！！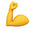 绿洲 刘宪华Henry-Lau的微博视频 转发理由:转发微博 (English Translation: #2020 #Wash your hands frequently and wear a mask. 2020 will definitely get better! Come on!!! 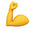
”

Table 2 summarizes the top 10 hashtags for each microblogging platform. To the extent that hashtags connect social media to a topic and make it easier to discover posts on a particular topic, these provided yet another indication of where the conversation on social media was directed during that period. Similar to the most frequent words, the hashtags largely converged, although they emphasized themes intended to bolster and galvanize the public’s fight against the virus. Further, although Twitter highlighted Hong Kong, in reference to the prodemocracy protests, Weibo hashtags did not register the topic in its top 10.

**Figure 1:**
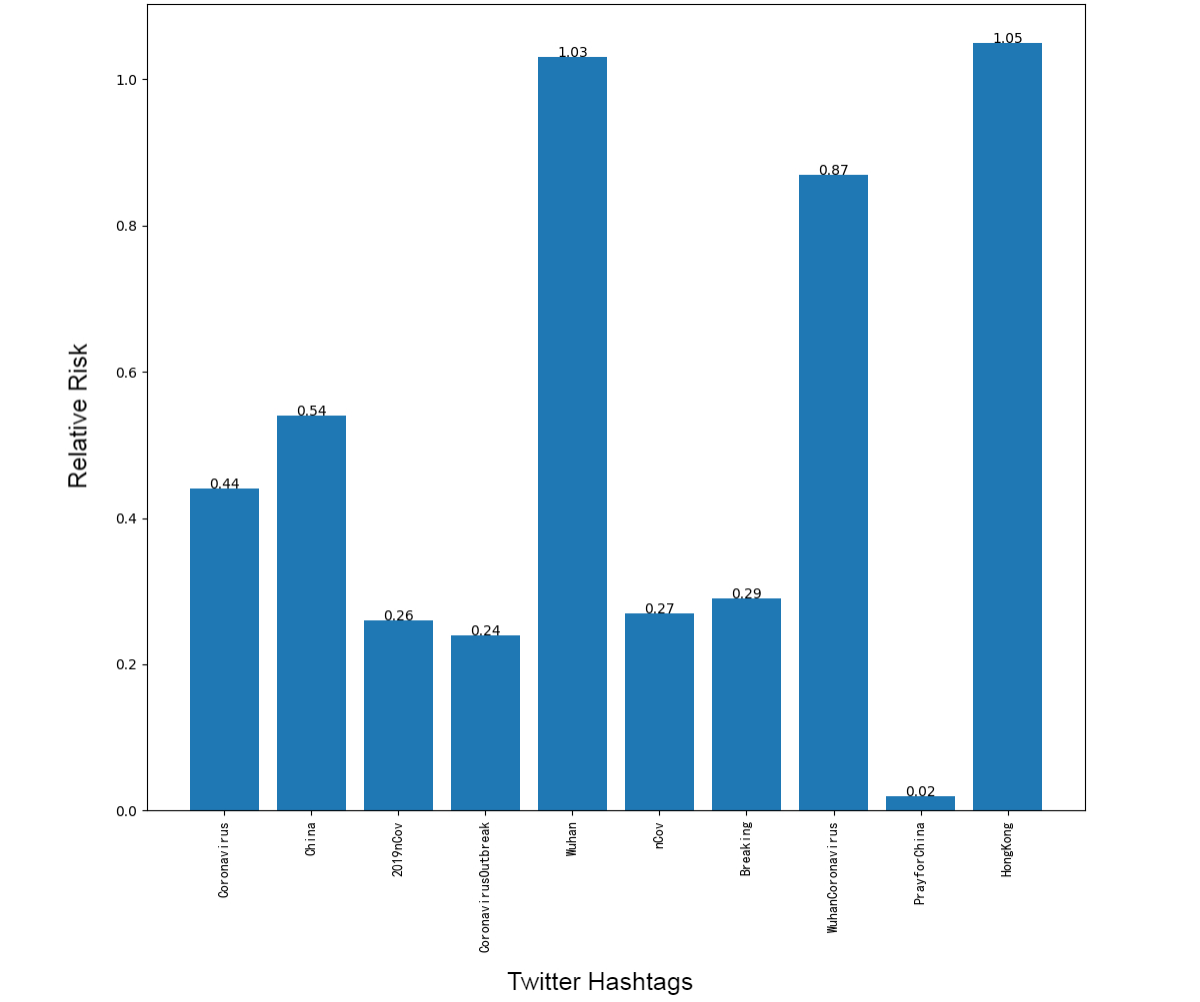
RR of Twitter hashtags (batch 1). RR: relative risk.

**Figure 2:**
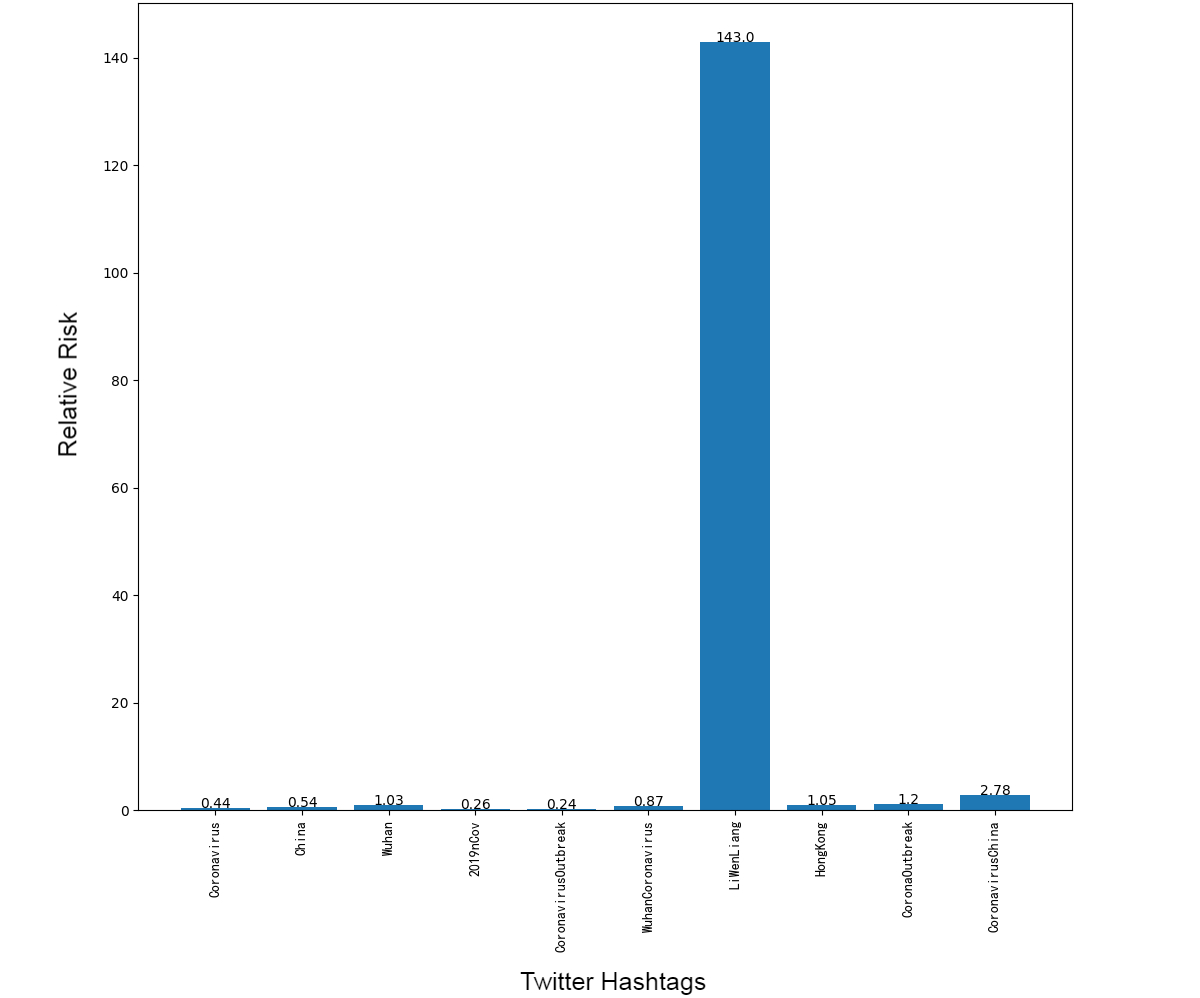
RR of Twitter hashtags (batch 2). RR: relative risk.

**Figure 3:**
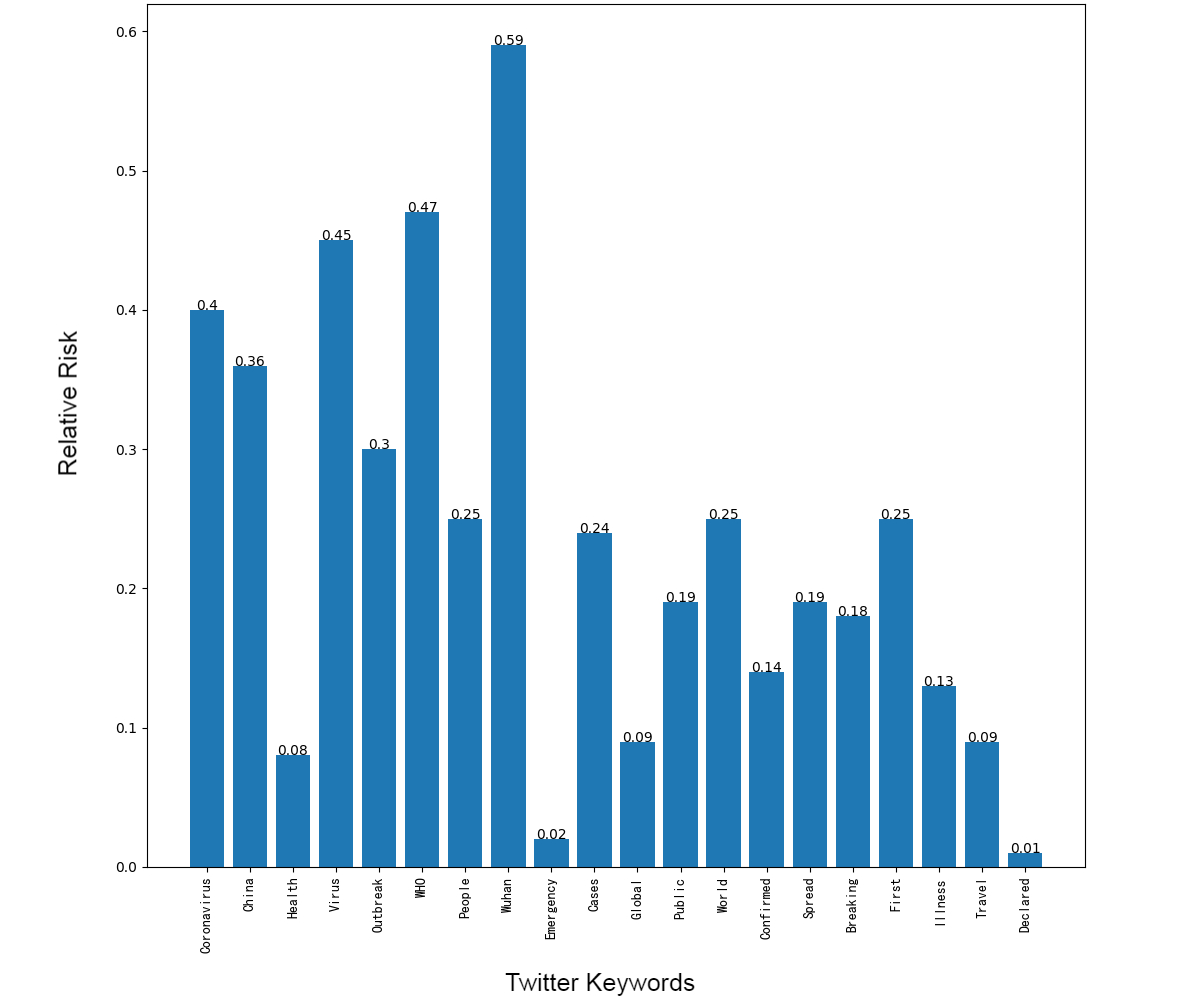
RR of Twitter keywords (batch 1). RR: relative risk.

**Figure 4:**
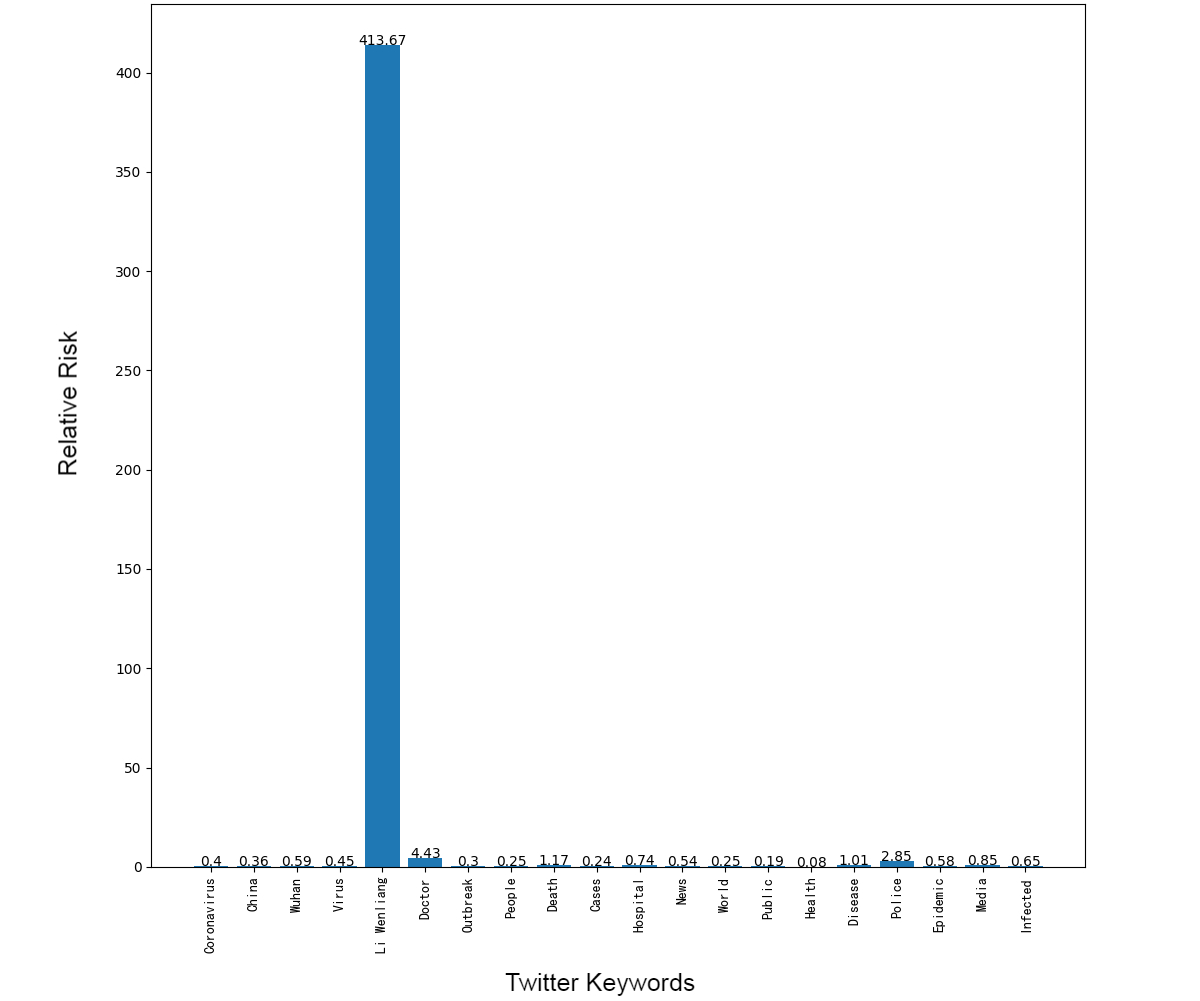
RR of Twitter keywords (batch 2). RR: relative risk.

**Figure 5:**
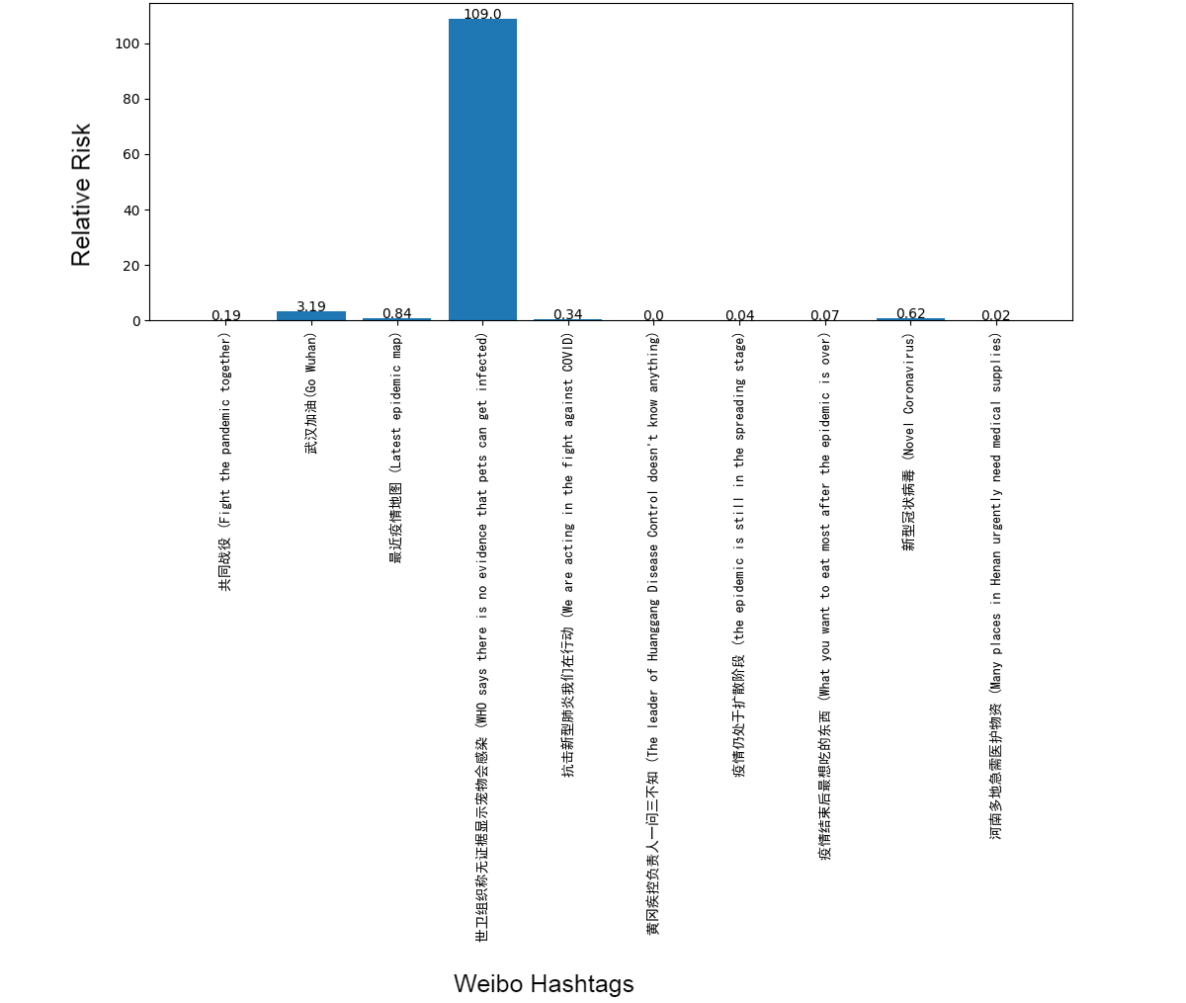
RR of Weibo hashtags (batch 1). RR: relative risk.

**Figure 6:**
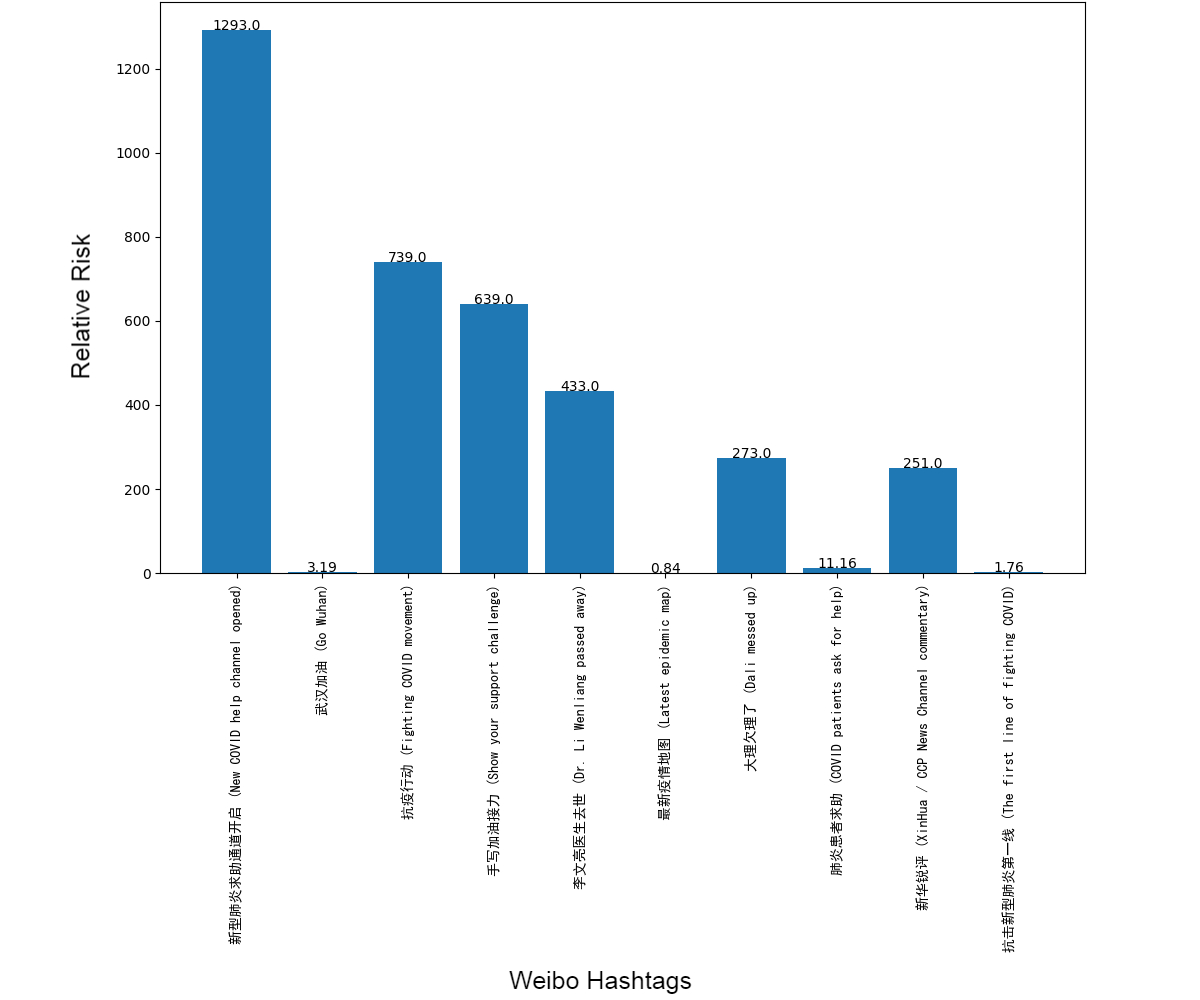
RR of Weibo hashtags (batch 2). RR: relative risk.

**Figure 7:**
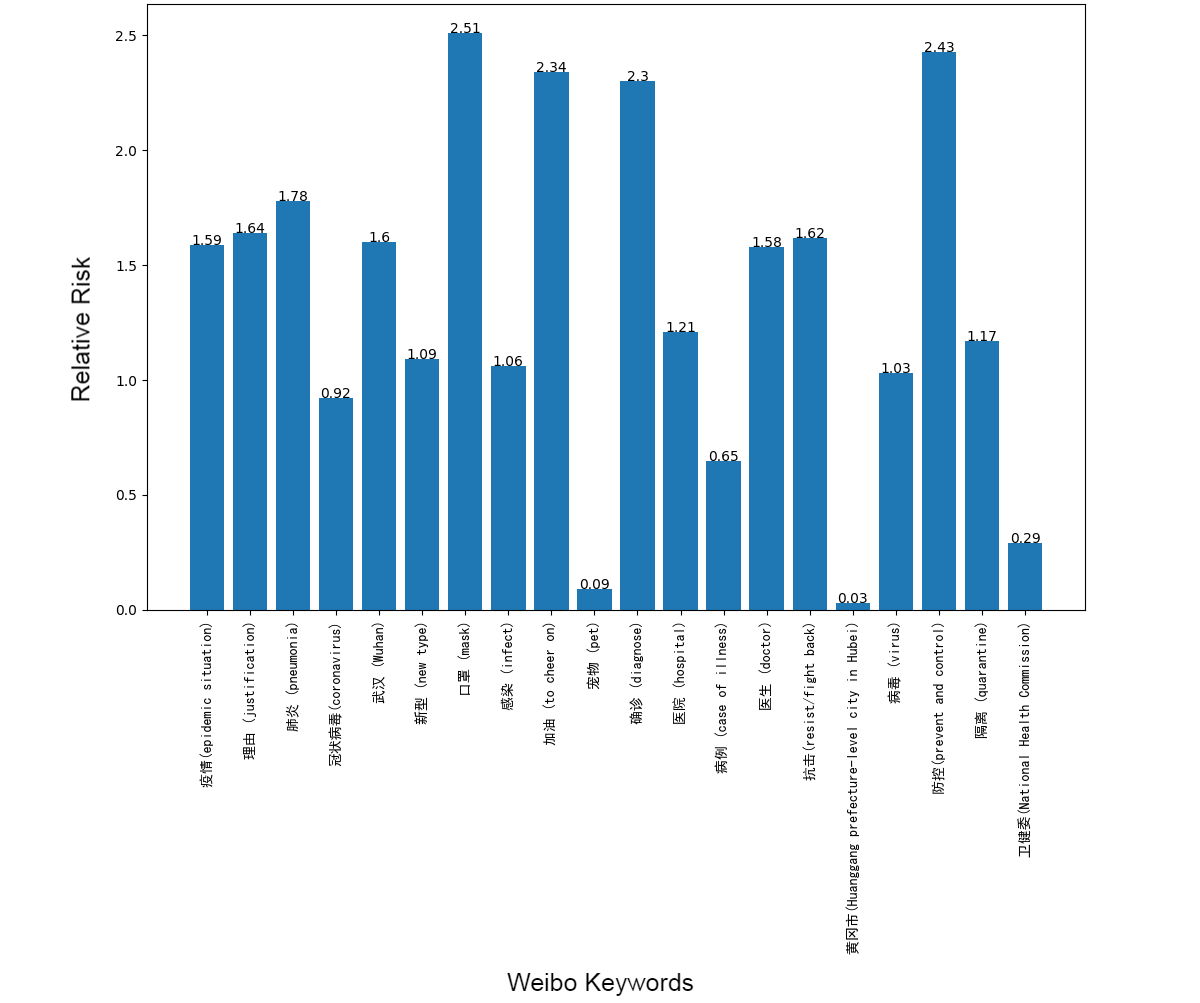
RR of Weibo keywords (batch 1). RR: relative risk.

**Figure 8:**
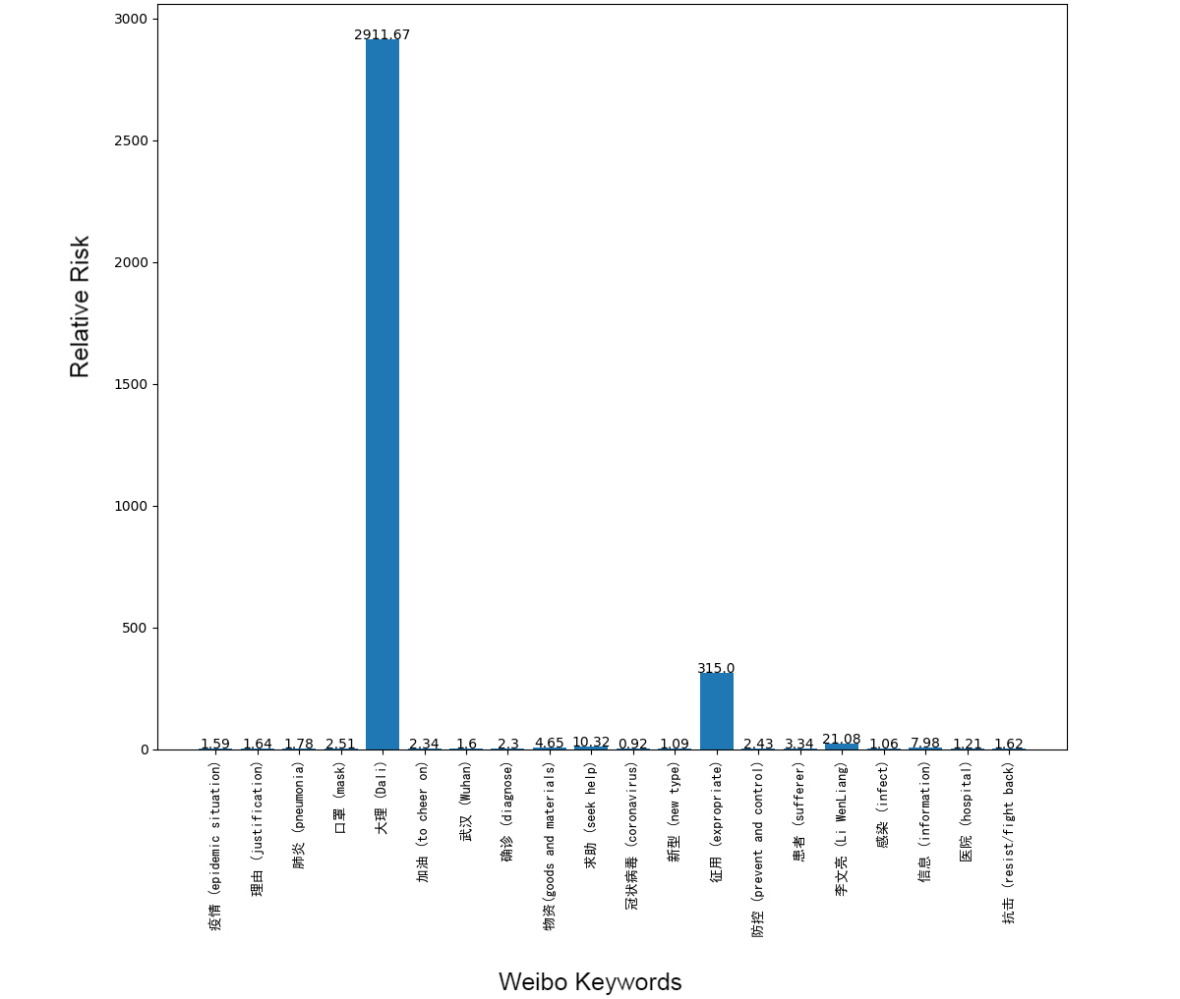
RR of Weibo keywords (batch 2). RR: relative risk.

Next, we addressed the RR based on the prevalence of topics between the 2 platforms, showing the frequency of posts on the pandemic over the 1-week period. Although our research design could not address self-moderation that likely occurs, in part, because individuals anticipate that certain posts will be removed and choose not to post certain material at all, it did at least gauge the moderation that took place over the week under study. Figures 1-8 show the RR computation for the top 20 most frequent keywords and the top 10 most frequent hashtags on both platforms across the 2 batches (January 30-31, 2020, and February 6-7, 2020). We found that the keyword with the highest RR (trending) was Li Wenliang for Twitter and Dali for Weibo.

Based on the random selection of 5%-7% of tweets that we manually coded, we found that most content focused news of the outbreak around the world and a growing number of COVID-19 cases across both batches. One representative tweet stated, “Breaking: There are 6 cases of coronavirus in the U.S., says @cdcgov. 1 person to person case has been confirmed in Chicago. CDC says this is a ‘very serious public health situation.’ They expect more cases. CDC is not recommending the general public wear face masks, as of now.” Misinformation was low on both sites, although it was comparatively higher on Twitter than on Weibo. We found that 1.1% of tweets from Twitter contained misinformation on COVID-19, with 5 (0.7%) of 746 tweets after discarding non-English posts in batch 1 and 6 (2.8%) of 211 tweets after discarding non-English posts in batch 2, compared to 0.3% on Weibo, with 1 (0.4%) of 279 posts in batch 1 and 1 (0.2%) of 441 posts in batch 2—a higher level of misinformation by a factor of almost 4 in the 1-week period on Twitter compared to Weibo.

**Figure 9:**
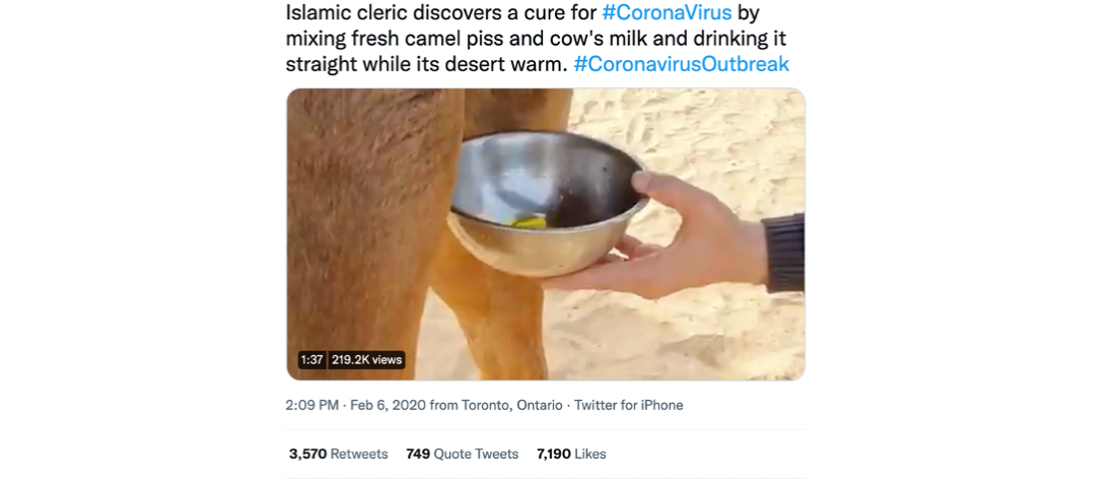
Screenshot of a public tweet that contains COVID-19 misinformation from batch 2.

By comparison with misinformation outside the domain of public health, Twitter reported that 0.3% of election-related tweets were flagged as misinformation [24]. Given the large volume of tweets posted on these topics, whether the election or coronavirus), and the tendency of users to engage with misinformation tweets more than accurate ones [25], the rate is notable. Textbox 1 outlines the various tweets that include misinformation found in both batches, and Figure 9 shows a screenshot illustrating an example of Twitter misinformation.

**Figure 10:**
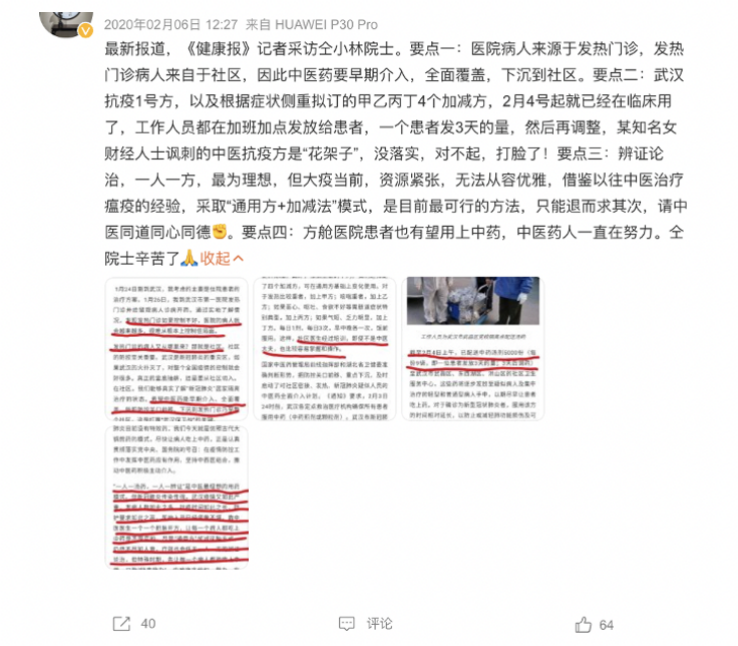
Screenshot of a public Weibo post that contains COVID-19 misinformation from batch 2. This post specifically discusses an “antiepidemic” prescription in treating those infected with COVID-19.

Misinformation was comparatively lower on Weibo, as seen in Table 3. Figure 10 shows an illustrative case of misinformation on the site. Of course, we cannot eliminate the possibility that more misinformation existed but was just removed quickly. Indeed, tweets themselves pointed to evidence of active moderation on Weibo, with 1 tweet stating that “the two trending topics censored by Weibo tonight: #wuhan government owes Dr. Li Wenliang an apology #we want freedom of speech #both had tens of thousands of views before disappearing into this dark night.”

**Misinformation tweets.**

**Batch 1 (5 tweets)**

- “#coronavirus possibilities:
  it is fear porn
  this is a vaccine scam
  this is a bio weapon leaked out but will be contained with a vaccine scam
  the chinese lab fucked up and let a bio weapon out they cannot stop now
  this is an illuminati depopulation plot

- “given that it's a global problem, the fact that the coronavirus only has around 10k confirmed cases and a 2% fatality rate means that you are more likely to get into a car accident than ever being influenced by this. despite that, the number of anti-chinese comments is crazy.”
- “deeply ridiculous: ‘indian government slammed for recommending homeopathy for coronavirus prevention’ https://t.co/stxcir5n2v what is the harm of tolerating pseudoscience? sigh.”
- “here we go, this will be trump's fault because of ‘climate change’ u.n. agency declares global emergency over virus from china: https://t.co/dyyedmdthr via @aol”
- “conspiracy theories surrounding #coronavirus as a lab made bioweapon somehow reminded me of commercial classic #7aumarivu of 2011 . this was reminded again by a friend during a conversation today. #arm was a visionary director indeed! #suriya https://t.co/vbiptn4wto”

**Batch 2 (6 tweets)**

- “islamic cleric discovers a cure for #coronavirus by mixing fresh camel piss and cow's milk and drinking it straight while its desert warm. #coronavirusoutbreak https://t.co/gypis0z1wf”
- “made in china to destroy canada”
- “something tells us, if anyone wants to find out #whatreallycaused the coronavirus pandemic that has infected thousands of people in china and around the globe, they should probably pay dr. peng a visit. dr peng can be reached at peng.zhou@wh.iov.cn, his phone# is 87197311 zh https://t.co/kzhlotnyjl”

- “cave full of bats in china identified as source of virus almost identical to the one killing hundreds today”
- “worst part about coronavirus is how it makes you super-paranoid when you get sick yrself. i'm clearly coming down w/a sinus infection, and obviously it has nothing to do w/that but man...on the other hand, i wonder if that chinese wuhan bat soup i had last week was a bad idea.”
- “sf officials urge public to attend lunar new year celebration, say there's no coronavirus threat. story by”

*Note*: Misinformation was coded based on a 5%-7% random sample of the initial 1% random sample, yielding 746 tweets for batch 1 and 211 tweets for batch 2.

**Table 3:** Misinformation posts (Weibo)a.

Language	Batch 1 (1 Weibo post)	Batch 2 (1 Weibo post)
Chinese	免疫力。近期的新型冠状病毒引起了社会的广泛关注，人们普遍认为抵抗力好的人，被传染的机率就小，而这与免疫力有很大的关系。免疫力是人体自身的防御机制，是人体识别和消灭外来侵入的任何异物（病毒、细菌等）、处理衰老、损伤、死亡、变性的自身细胞以及识别和处理体内突变细胞和病毒感染细胞的能力。中医认为所有的好的东西，比如人们常说的免疫力、抵抗力都称之为“正气”，一切导致疾病的因素，称为“邪气”。当人的正气充足的时候，就不被邪气所侵犯，所以《黄帝内经》曰：“正气内存，邪不可干，邪之所凑，其气必虚” 。也就是说，当你正气充足的时候，一切致病因素的邪气就拿你没办法，不可能侵犯你。而你得病的时候，一定是正气虚的时候。因此，中医强调正气一定要充足，那么免疫力就会比其他人好，患病的几率也会下降很多	最新报道，《健康报》记者采访仝小林院士。要点一：医院病人来源于发热门诊，发热门诊病人来自于社区，因此中医药要早期介入，全面覆盖，下沉到社区。要点二：武汉抗疫1号方，以及根据症状侧重拟订的甲乙丙丁4个加减方，2月4号起就已经在临床用了，工作人员都在加班加点发放给患者，一个患者发3天的量，然后再调整，某知名女财经人士讽刺的中医抗疫方是“花架子”，没落实，对不起，打脸了！要点三：辨证论治，一人一方，最为理想，但大疫当前，资源紧张，无法从容优雅，借鉴以往中医治疗瘟疫的经验，采取“通用方+加减法”模式，是目前最可行的方法，只能退而求其次，请中医同道同心同德。要点四：方舱医院患者也有望用上中药，中医药人一直在努力。仝院士辛苦了转发理由:转发微博
English translation	Immunity. The recent novel coronavirus has aroused widespread concern in society. It is generally believed that people with good resistance have a smaller chance of being infected, and this has a lot to do with immunity. Immunity is the body's own defense mechanism. It is the body's ability to recognize and eliminate any foreign objects (viruses, bacteria, etc.) that invade from the outside, deal with aging, damage, death, and degeneration of its own cells, as well as recognize and process mutant cells and virus-infected cells in the body. Chinese medicine believes that all good things, such as immunity and resistance, are called “zhengqi”(positive energy), and all factors that cause diseases are called “xieqi” (evil energy). When a person's zhengqi is sufficient, he will not be invaded by evil spirits, so the “Huangdi Neijing” (this is a traditional Chinese medicine book) says: “If there is a zhengqi (positive energy), xieqi (evil energy) can not interfere, if evil is combined, its energy will be empty.” In other words, when your zhengqi is sufficient, all the xieqi of the pathogenic factors can do nothing, and it is impossible to invade you. And when you get sick, it must be a time of deficiency of zhengqi. Therefore, Chinese medicine emphasizes that there must be sufficient zhengqi, then the immunity will be better than other people, and the chance of illness will be much lower.	The latest report, a reporter from “Health Daily” interviewed Academician Tong Xiaolin. Key point 1: Hospital patients come from fever clinics, and fever clinic patients come from the community. Therefore, Chinese medicine should intervene early, fully cover, and sink into the community. Point # 2: No. 1 party Wuhan fight against SARS, according to the symptoms and focus on ABC D plus or minus four parties prepared, February 4 onwards has been in clinical use, and the staff are issued to patients in overtime, made a 3 patients The amount of the day, and then adjust, a well-known female financial person ridiculed the traditional Chinese medicine anti-epidemic prescription is “flowery”, not implemented, sorry, face! Point 3: Syndrome differentiation and treatment, one person for one party is the most ideal. However, the current pandemic, resources are tight, and it is impossible to be calm and elegant. Learning from the past experience of Chinese medicine in treating plagues, adopting the “general prescription + addition and subtraction” model is the most feasible method at present Can retreat to the second best, please Chinese medicine fellows with one heart and one mind. Point 4: Patients in Fangcang shelter hospitals are also expected to use Chinese medicine. Chinese medicine practitioners have been working hard. The same academician worked hard. Reason for forwarding: forward Weibo

^a^Misinformation was coded based on a 5%-7% random sample of the initial 1% random sample, yielding 279 posts for batch 1 and 441 posts for batch 2. Weibo increased the post text limit to 2000 characters. However, posts longer than 140 characters are truncated on the platform, but users can click a “see entire” text button to unfold the rest of the post.

## Discussion

### Principal Findings

We found that across 2 widely used platforms in and outside China, Weibo and Twitter, the nature of discourse converges to a considerable degree, with the platforms both being used to exchange information about the transmission, prevention, and impacts of the COVID-19 pandemic. Similar to Wang et al [26], however, we found far more of a positive, cheerleading valence on Weibo compared to Twitter, with the Chinese microblogging site frequently emphasizing the community fight against the virus in ways that are not observable on Twitter. Correspondingly, as with Lu et al [17], we did see the presence of topics that might be seen as implicating the regime in a negative light, such as the references to the whistleblower doctor, but those were balanced out with the supportive content referenced above. Twitter users, corroborating the finding of Deng et al [27], were attentive to economic implications of the pandemic compared to the virtual nonexistence on Weibo. In terms of misinformation, Weibo had comparatively less misinformation than Twitter, which corroborates a previous analysis of relative cross-platform differences in the context of Ebola that showed less misinformation on the Chinese site [18].

Taken together, our research makes several contributions to the understanding of cross-platform, cross-national content exchange and misinformation across in the context of COVID-19. First, although scholars have studied misinformation in a political context [28], in previous medical epidemics [29], and increasingly in the COVID-19 context [30], comparative study is more limited. Second, a better understanding of misinformation matters in a public health context because it has implications for whether individuals can make meaningful choices about policies, for example, the risks and benefits of complying with public health guidance [6]. Third, the dissemination of misinformation—because of its connection with a range of behaviors such as anxiety, self-prescription of medication and treatments, erosion of trust in government authorities [31], and lower compliance rates on public health measures such as social distancing measures [32]—foreshadows likely public health outcomes. Thus, a closer scrutiny of both patterns of discussion on social media and the presence of misinformation has important implications for anticipating the future course of a virus that has claimed millions of lives. Finally, understanding COVID-19 content in a cross-national context helps shed additional light on differences in algorithms and interventions that Weibo versus Twitter use to structure content [15], while also informing potential countermeasures for online misinformation, such as flagging, correcting, or removing online content [10].

### Limitations

Our study does have limitations. First, we note that our results are not generalizable due to the small sample size of tweets and posts that were reviewed, which was based on a 1% random sample of content. Second, we compared posts on Weibo and Twitter at the same point in time in the interest of internal validity, based on WHO’s declaration of a global health emergency, which provided a common baseline. We recognize, however, that the arc of COVID-19 was different in China (expressed on Weibo) than outside China (Twitter), which may have affected the nature of posts and the public interest or tolerance for posting misinformation. On January 23, 2020, for example, Wuhan’s 11 million residents had been cordoned off from the rest of the country, speaking to the intensity of the virus already by the time of the WHO declaration. By contrast, the first COVID-19 death in Europe was not reported until February 12 and, New York City schools closed on March 15 [33]. Future research should compare potential levels of misinformation at various points during the pandemic in different countries beyond the 1-week mark of the WHO announcement.

Although there were only a few posts containing misinformation across the Twitter and Weibo batches in our study, we acknowledge that misinformation comprises a small percentage of the overall content based on our manual coding. For reference, Fung et al [18] identified 6 tweets and 2 tweets in batch 1 and batch 2, respectively, as alternative health information on Twitter, and 11 posts and 3 posts in batch 1 and batch 2, respectively, on Weibo. These are not large numbers by any means for both platforms, which is similar to our study’s single-digit posts, which we identified as misinformation. The 1% random samples of tweets and Weibo posts facilitates a fair way of assessing the representative content based on various categories and minimizes biases. This study’s findings are mostly explanatory in nature regarding the level of misinformation found on both platforms during the 1-week period. However, additional research could replicate our study with different 1% random samples of tweets and Weibo posts and examine whether there is consensus or contrasting findings.

Further, although our analysis was agnostic about the position of WHO, social media platforms, and the Centers for Disease Control and Prevention (CDC) that more aggressive moderation was warranted, given the public health crisis, we acknowledge the possibility of overreach. Future studies might also engage with normative questions about the potential for overreach when it comes to content moderation, considerations about whether organizations such as WHO should be endorsing control of information, comparison of COVID-19 content on both platforms with longer periods, and the inverse of our study, which is to analyze posts or accounts that were removed due to misinformation but ultimately found to be accurate and permissible.

### Conclusion

In May 2020, WHO observed that “managing the infodemic is a critical part of controlling the COVID-19 pandemic: it calls on Member States to provide reliable COVID-19 content, take measures to counter mis- and disinformation and leverage digital technologies across the response” [34]. We showed that Twitter and Weibo, the 2 most widely used microblogging platforms in the United States and China, respectively, have carried out information management in different ways. Perhaps most notable is not the reliability of content—both had low levels of misinformation—but rather the absence of certain topics, such as WHO, Hong Kong, and death, as well as the tendency of Weibo posts to provide societal cheerleading, a phenomenon absent on the US-based equivalent. One limitation of our study is the small sample size of the overall COVID-19 content on Twitter and Weibo during this 1-week period. However, we invite and encourage future research to incorporate a larger sample size of tweets and posts and examine longer periods on this important topic.

## Abbreviations

API: Application Programming Interface
RR: relative risk
WHO: World Health Organization
